# Improving handover competency in preclinical medical and health professions students: establishing the reliability and construct validity of an assessment instrument

**DOI:** 10.1186/s12909-021-02943-x

**Published:** 2021-10-02

**Authors:** Meghan Michael, Andrew C. Griggs, Ian H. Shields, Mozhdeh Sadighi, Jessica Hernandez, Chrissy Chan, Mary McHugh, Blake E. Nichols, Kavita Joshi, Daniel Testa, Sonika Raj, Richard Preble, Elizabeth H. Lazzara, Philip E. Greilich

**Affiliations:** 1 Department of Anesthesiology and Pain Management, University of Texas Southwestern Medical Center, 5323 Harry Hines Blvd, Mail Code 9068, Dallas, TX 75390 USA; 2 Department of Human Factors and Behavioral Neurobiology, Embry-Riddle Aeronautical University, 1 Aerospace Blvd, Daytona Beach, FL 32114 USA; 3 Office of Quality, Safety, and Outcomes Education, University of Texas Southwestern Medical Center, 5323 Harry Hines Blvd, Dallas, TX 75390 USA; 4 Department of Undergraduate Medical Education, University of Texas Southwestern Medical Center, 5323 Harry Hines Blvd, Dallas, TX 75390 USA; 5 Department of Emergency Medicine, University of Texas Southwestern Medical Center, 5323 Harry Hines Blvd, Dallas, TX 75390 USA; 6 Department of Pediatrics, University of Texas Southwestern Medical Center, 5323 Harry Hines Blvd, Mail Code 9063, Dallas, TX 75390 USA

**Keywords:** Teamwork, Handovers, Measurement, Reliability, Validity

## Abstract

**Background:**

As part of the worldwide call to enhance the safety of patient handovers of care, the Association of American Medical Colleges (AAMC) requires that all graduating students “give or receive a patient handover to transition care responsibly” as one of its Core Entrustable Professional Activities (EPAs) for Entering Residency. Students therefore require educational activities that build the necessary teamwork skills to perform structured handovers. To date, a reliable instrument designed to assess teamwork competencies, like structured communication, throughout their preclinical and clinical years does not exist.

**Method:**

Our team developed an assessment instrument that evaluates both the use of structured communication and two additional teamwork competencies necessary to perform safe patient handovers. This instrument was utilized to assess 192 handovers that were recorded from a sample of 229 preclinical medical students and 25 health professions students who participated in a virtual course on safe patient handovers. Five raters were trained on utilization of the assessment instrument, and consensus was established. Each handover was reviewed independently by two separate raters.

**Results:**

The raters achieved 72.22 % agreement across items in the reviewed handovers. Krippendorff’s alpha coefficient to assess inter-rater reliability was 0.6245, indicating substantial agreement among the raters. A confirmatory factor analysis (CFA) demonstrated the orthogonal characteristics of items in this instrument with rotated item loadings onto three distinct factors providing preliminary evidence of construct validity.

**Conclusions:**

We present an assessment instrument with substantial reliability and preliminary evidence of construct validity designed to evaluate both use of structured handover format as well as two team competencies necessary for safe patient handovers. Our assessment instrument can be used by educators to evaluate learners’ handoff performance as early as their preclinical years and is broadly applicable in the clinical context in which it is utilized. In the journey to optimize safe patient care through improved teamwork during handovers, our instrument achieves a critical step in the process of developing a validated assessment instrument to evaluate learners as they seek to accomplish this goal.

## Background

Handover of patient care, defined as the transition of information and responsibility from one individual or team to another [1, 2], is a critical time in a patient’s medical journey. Handovers are high-frequency, error-prone events that have been implicated in nearly 80 % of serious adverse events [3]. Given the criticality of handovers, organizational bodies have put forth mandates to strengthen handovers. The World Health Organization [4, 5] and Joint Commission [6] have mandated a more structured process to mitigate the risk of communication failures on patient safety. Although there is meta-analytic evidence that suggests structure within handovers improves patient outcomes [7], the specific structure is left to individual institutions to adopt.

Governing bodies for undergraduate [8–10] and graduate [11] education identified handovers as a core professional skill that future healthcare workforce must be prepared to perform. Accredited training programs must prepare and validate a healthcare trainee’s behavioral competencies in conducting safe patient handovers. These teamwork behaviors are essential non-technical skills required to achieve highly reliable interprofessional communication in a variety of ambulatory and inpatient settings [12, 13].

Despite these educational mandates, there is little guidance on when training in teamwork competencies should begin. Currently, early exposure of undergraduate learners to the skills needed for interprofessional patient handovers is largely ignored and reflects a significant gap in pre-clinical education [2, 14–16]. Emerging evidence suggests that introduction of interprofessional educational activities in preclinical learners can improve attitudes and skills needed for team-based communication during handovers [17–19]. In addition, this strategy helps to mitigate the risk of introducing this core entrustable professional activity during clinical rotations, which can be unpredictable and prone to inconsistency, being untestable, and frequently unsustainable [19–22]. Given this background, an essential component of a longitudinal curriculum designed to improve competencies required for handovers is a psychometrically sound assessment instrument that is appropriate for preclinical learners with minimal or no clinical experience [22–24].

The development of a reliable and valid instrument capable of assessing constructs critical to safe patient handovers is essential. Such an instrument would not only permit the assessment of learners’ proficiency in the required behaviors, but also, secondarily, the curriculum itself. Ideally, an assessment instrument needs to be generalizable and subjected to rigorous methodological design [25–27]. Despite the high number of available teamwork and communication-focused interventions [2, 28–31], a literature review found that assessment instruments for discrete handover competencies were rare and had widespread heterogeneity depending on specialty, profession, handover context, and institution [32]. In fact, consensus among systematic reviews suggests ongoing gaps in available evidence regarding high-quality assessment instruments of competency-based training in handovers, communication behaviors, and teamwork behaviors [33–38].

Previous systematic reviews by Gordon, et al. [2, 34], found reliability and/or validity of assessment instruments for handover competencies were reported in only a few studies even though conclusions on their efficacy and usability were common. Muller, et al. [16] reported similar findings in handover instruments used to assess educational curricula designed to teach structured communication using the “Situation-Background-Assessment-Recommendation” (SBAR) handover tool [39]. In studies reporting reliability or validity data, Davis, et al. [38] found evaluator training, experimental conditions, and study bias were poorly controlled. The available evidence underscores the need to develop reliable instruments for assessing curricula designed to teach teamwork and communication in pre-clinical learners.

The purpose of this study is to establish the reliability and construct validity of an assessment instrument capable of evaluating communication competencies for handovers (i.e., structured communication, closed-loop communication, and question clarification) in preclinical medical and health professions students. We use the clinical handover as a pedagogical vehicle to accomplish assessment of learners and leverage the SBAR framework to enable pre-clinical learners to engage in a clinical simulation targeting communication skills during handovers.

## Methods

### Study design and setting

We developed a curriculum to teach preclinical students with limited to no clinical experience multiple competencies as they relate to handovers (i.e., structured communication, closed-loop communication, and asking clarifying questions). The curriculum assumed that learners had three semesters of didactic basic science education but no prior clinical science training. The curriculum also involved prereading, videos, and activities that were facilitated by clinical educators and hosted in Microsoft Teams (Microsoft Corporation, version 4.2.4.0) [40] in which groups of preclinical students acted as handover senders and receivers for four simulated patient scenarios. Students reviewed the four patient scenarios and performed simulated handovers for each patient during the activity. Audiovisual recordings of these handovers were collected and reviewed by multiple raters. The raters utilized a seven-item behavioral assessment instrument developed by our team to rate students’ performance based on the degree to which they handed over pertinent clinical information and whether they leveraged clarifying questions and closed-loop communication during the handover. Data collected by multiple raters reviewing these recordings were collated and analyzed for inter-rater reliability and construct validity.

### Participants

During the handover activity, multiple groups of four to six interprofessional students performed handovers for each of the four simulated patient scenarios facilitated by a clinical educator. Each handover occurred between one sender and one receiver assigned by the educator, while the rest of the team played an observer role. In total, 192 handovers were recorded from a sample of 229 medical students and 25 health profession students who participated in the activity (*N* = 254 students). After the handover activity, a team of five raters reviewed recordings of the students’ handovers to evaluate their performance (*N* = 180 reviewed handovers).

### Materials

#### Patient scenarios

The simulated patient scenarios (Scenario A, B, C, and D) contained all the information to be handed over during the activity. The patient scenarios were pre-recorded in a simulation center. Each scenario featured a patient describing their symptoms, a physician assessing the patient and asking them questions, and a nurse checking the patient’s vitals. Students were assigned to review either scenarios A and B, or C and D before joining the handover activity and were instructed to utilize SBAR as a structured communication tool to organize the information from the patient scenarios during their handovers. Table 1 provides a summary of the patient scenarios including the patients’ gender, age, and chief complaint.

**Table 1:** Patient scenarios

Scenario Label	Patient Gender	Patient Age	Chief Complaint
A	Male	50	Shortness of breath
B	Male	75	Foul smelling urine
C	Female	55	Hypertension
D	Female	35	Acute onset headache

#### Behavioral measure

To facilitate the raters’ measurement of students’ handover performance during the activity, we developed a behavioral assessment instrument with items related to three handover communication competencies: structured communication via Situation, Background, Assessment, and Recommendation (SBAR; 4 items), closed-loop communication (2 items), and asking clarifying questions (1 item). The inclusion of these seven items was primarily based on three considerations.

The first consideration centered on the first four items of the tool (i.e., the items related to SBAR). SBAR was utilized due to its substantial empirical support as well as it being widely used and recommended [16, 41]. To elaborate, SBAR has a long history across a variety of countries, domains, and provider types. In fact, a systematic review of handoff protocols found that a majority of handoff mnemonics were leveraging the SBAR structure [41]. Additionally, there is evidence to suggest that SBAR is associated with enhanced perceptions as well as patient outcomes [42]. Due to such evidence, SBAR is recognized as an effective communication tool by The Joint Commission, the Agency for Healthcare Research and Quality, Institute for Healthcare Improvement, and the World Health Organization [42] as well as the Royal College of Physicians [43]. Such recommendations are rooted in the notion that structured communication enhances shared understanding and the potential to mitigate memory lapses.

The second consideration focused on closed-loop communication, which relates to the next two items on the tool. Closed-loop communication has been widely touted as a useful component within effective teamwork (e.g., Salas, et al. [44, 45]) and even patient care [44, 46]. As further support, a recent narrative review stated that closed-loop communication is one of the most useful strategies for strengthening communication for specific medical teams [47]. Similar to structured communication, closed-loop communication fosters accuracy and ameliorates potential errors.

The last consideration targeted the remaining item – asking clarifying questions. That item was included due to its criticality in patient care and even handovers specifically [48–50]. As such, the Joint Commission and the Agency for Healthcare Research and Quality have explicitly stated that asking questions should be formally incorporated during handovers [51, 52]. These mandates are grounded in the idea that asking questions strengthens resiliency [53]. Table 2 provides a list of all the items on this behavioral assessment instrument. The three aforementioned considerations served as the theoretical basis for the development of the seven items in our assessment instrument.

**Table 2:** Behavioral assessment instrument items

Associated Competencies	Items
Structured Communications	1. Sender described *situation* succinctly
	2. Sender provided additional *background* info
	3. Sender offered *assessment* based on situation and background
	4. Sender stated clear *recommendation* about what should happen
Closed-Loop Communications	5. Receiver repeated what they heard (not just “I understand”)
	6. Sender clearly confirmed or corrected the check back
Asking Clarifying Questions	7. Receiver asked clarifying questions from sender when receiving handover

Each item could receive one of three scores based on pre-defined behavioral anchors: No/Never, Sometimes/Somewhat, or Yes/Always. We developed a scoring guide to provide to the raters before reviewing any videos and later revised the guide to bolster inter-rater reliability. The scoring guide provided guidance concerning when each scoring option should be used for all items as well as the most pertinent information to be handed over for the structured communication items in each patient scenario. Students did not have access to the assessment instrument or the scoring guide. However, detailed instructions and informational material on both structured handovers and team competencies, supplemented with examples, were shared with the students in the format of pre-reading documents and videos. Table 3 provides a summary of this scoring guide.

**Table 3:** Summary of scoring guide

Competency	Item Number(s)	No/Never Scoring Requirements	Sometimes/Somewhat Scoring Requirements	Yes/Always Scoring Requirements
Structured Communications	1–4	*No* pertinent SBAR information handed over	A *portion* of pertinent SBAR information handed over	*All* pertinent SBAR information handed over
Closed-Loop Communications	5	Receivers only stated “I understand” or failed to repeat any information from *any components of SBAR*	Receivers repeated at least one piece of information from *at least one component of SBAR*	Receivers repeated at least one piece of information from *each component of SBAR*
	6	Senders fail to confirm or correct *any* the information given in the checkback	Senders confirmed or correct *a portion* of the information given in the checkback	Senders confirmed or correct *all* information given in the checkback
Asking Clarifying Questions	7	Receivers asked *no* clarifying questions	Receivers asked at least *one* clarifying question	Receivers asked at least *two or more* clarifying questions

### Procedure

One hour before the handover activity, students were provided with a Microsoft Teams meeting invitation and links to access the patient scenarios to be handed over during the activity via email. Students were assigned two patient scenarios to review (A and B, or C and D) before using the provided link to join the handover activity in Microsoft Teams. After joining the activity, students were assigned to act as either a handover sender for one of the patients in the scenarios they were instructed to review or a receiver for one of the patients that they did not review. Each handover had one sender and one receiver. Recordings of these handovers were stored in a secure, network-enabled repository for later analysis.

We developed a scoring guide to aid raters in assessing students’ performance during the activity when reviewing the recordings. We then assembled a multidisciplinary team of five raters from varying clinical backgrounds, including faculty from the Emergency Medicine and Pediatrics departments as well a medical student (Dean’s scholar), that were familiar with SBAR handovers and the clinical competencies being assessed. We held a meeting with the raters to describe the handovers that were performed as well as the scoring guide and behavioral assessment items that were developed. Following this meeting, the raters were tasked with reviewing twelve handovers across three video recordings to serve as training before reviewing the full sample of videos. Inter-rater reliability statistics were calculated based on the data collected from these three videos, and the scoring guide was revised for clarity based on rater feedback. Next, the raters were tasked with reviewing the remaining videos in pairs such that each video was reviewed by at least two different raters. Finally, once all videos were reviewed by the team of raters, their data were collated, and their inter-rater reliability was assessed.

### Statistical analysis

Following training, data from the team of five raters were collated and imported into IBM’s SPSS software (Version 27) [54] for analysis. We identified instances where all raters were in total agreement or at least one rater was in disagreement for each item across handovers under analysis and calculated percent agreement by comparing the proportion of each response. As all raters reviewed all subjects at this stage (i.e., handovers contained in videos) and their responses were recorded as categorical variables (i.e., No/Never, Sometimes/Somewhat, or Yes/Always), we used Fleiss’ kappa to assess inter-rater reliability. Fleiss’ kappa is a measure of inter-rater reliability that can be used with categorical data collected from two or more raters [55]. Table 4 provides common cut-off values denoted by Landis and Koch [56] that are applicable to Fleiss’ kappa.

**Table 4:** Kappa Value Interpretation Cut-Offs Provided by Landis and Koch [56]

Kappa Value	Interpretation
< 0	Poor Agreement
0.01–0.20	Slight Agreement
0.21–0.40	Fair Agreement
0.41–0.60	Moderate Agreement
0.61–0.80	Substantial Agreement
0.81-1.00	Almost Perfect Agreement

Once all videos were reviewed by the rating team, we collated and imported the data for the full sample of videos into SPSS to calculate percent agreement and overall measures of inter-rater reliability. At this stage, since not all raters reviewed all subjects (i.e., five raters for each training video and two raters per remaining video), we utilized the Krippendorff’s alpha SPSS macro developed by Andrew Hayes [57] to calculate an inter-rater reliability coefficient from the entire data set. Krippendorff’s alpha can be used with two or more raters and response categories, ordinal data, or missing data, and the same cut-off values provided in Table 4 can be applied to Krippendorff’s alpha [55]. We also utilized Cronbach’s alpha to serve as a secondary measure of reliability.

Prior researchers have noted that what constitutes acceptable inter-rater reliability based on these qualitative cutoffs is often debated [58, 59]. Krippendorff [60] has provided more conservative interpretations based on their work in content analysis (i.e., “rely only on variables with reliabilities above *α* = .8,” p. 241). Values below this cut-off are often retained in research, however, and what constitutes acceptable inter-rater reliability can vary depending on the hypotheses or research questions under observation [58]. Concerning healthcare research, McHugh [59] has argued that kappa values below 0.60 are unacceptable.

Following assessment of inter-rater reliability, we performed a confirmatory factor analysis (CFA) in IBM’s SPSS software (Version 27) [54] to establish a preliminary measure of this instrument’s construct validity. A single rating was determined for each item across handovers in this analysis using the mode of raters’ responses when possible. In instances where a mode response could not be calculated (i.e., for items in which two raters provided non-matching responses), a single rater’s data were used. As the items in this instrument were based on three distinct clinical competencies needed for effective handovers, a fixed number of three factors were selected a priori to be extracted in our analysis.

## Results

### Reliability

#### Results from rater training

We collated data from twelve handovers reviewed by the team of raters during training and calculated percent agreement and Fleiss’ kappa as preliminary measures of inter-rater reliability at this stage. From the handovers reviewed during training (i.e., three videos containing four handovers each, with each handover rated using seven items), the raters achieved 31.25 % agreement across the 84 items. We calculated Fleiss’ kappa and achieved a coefficient of 0.339, indicating fair agreement among the raters. Based on these data, we revised the scoring guide for clarity and tasked the five raters with reviewing the remaining videos in pairs.

#### Results from full assessment

Following training and the review of all remaining videos, we recalculated percent agreement and ran Krippendorff’s alpha to assess inter-rater reliability. Based on the data from the full sample of handovers (i.e., 45 videos containing 4 handovers each that were rated using 7 items), the raters achieved 72.22 % agreement across the 1,260 items. We calculated Krippendorff’s alpha and achieved a coefficient of 0.6245, indicating substantial agreement among the raters. We also calculated Cronbach’s alpha and achieved a coefficient of 0.63.

### Construct validity

As a preliminary measure of construct validity, we ran a CFA on the 7-items in our instrument that measured three competencies related to handovers: structured communications, closed-loop communications, and asking clarifying questions. We assessed the suitability of CFA prior to analysis; inter-item correlations between − 0.093 and 0.797 were observed, the overall Kaiser-Meyer-Olkin (KMO) measure was 0.552 with individual KMO measures between 0.359 and 0.977, and Bartlett’s test of sphericity was statistically significant (*p* < .001).

CFA revealed that three factors had eigenvalues of approximately one or greater which explained 27.7 %, 21.6 %, and 14.2 % of the total variance, respectively. Visual inspection of the scree plot also indicated that three factors should be retained. The three-factor structure explained 63.5 % of the total variance. We used a Varimax orthogonal rotation to support interpretability. Items regarding closed-loop communication had strong loadings on Factor 1, items concerning SBAR exhibited moderate to weak loadings on Factor 2, and the item regarding clarifying questions exhibited a moderate loading onto Factor 3. A summary of rotated factor loadings is presented in Table 5. These loadings demonstrate preliminary evidence of the orthogonal nature of constructs measured using the seven items in this instrument.

**Table 5:** Rotated factor matrix

Items	Factor		
	**1**	**2**	**3**
Sender clearly confirmed or corrected the check back	0.932		
Receiver repeated what they heard (not just “I understand”)	0.928		
Sender offered assessment based on situation and background		0.725	
Sender described situation succinctly		0.671	
Sender stated clear recommendation about what should happen		0.624	− 0.418
Receiver asked clarifying questions from sender when receiving handover			0.774
Sender provided additional background info		0.377	0.484

## Discussion

Our team has described an assessment instrument that demonstrates substantial agreement between raters as well as some preliminary evidence towards construct validity in discerning proficiency in the use of structured handover format as well as two communication competencies required for safe patient handovers. In addition to filling the void of reliable and construct valid assessments that address both structured handovers and teamwork competencies, this instrument addresses an important gap in preclinical medical education for teamwork competencies given the lack of psychometrically sound instruments for assessments that can be applied to the preclinical and interprofessional population.

In addition to filling the aforementioned gaps, our instrument offers many features that make it useful for evaluators and instructors. First, it is appropriate for learners of all levels, including those in their preclinical years of training. Because all learners throughout the medical education curriculum will need to demonstrate proficiency in handovers and teamwork, it is imperative that all learners are assessed with an appropriate, yet scientifically sound, instrument. Second, our instrument is generalizable to any clinical context. Because teamwork is necessary in any facet of care and handovers are conducted by every clinical role in any department, it is critical that learners are competent independent of clinical context. Third, our instrument is generalizable for any handover. Handovers are extremely heterogeneous; that is, the same information is not necessarily exchanged across contexts. For example, the information exchanged in a handover between two anesthesia providers during an anesthetic will contain very different pertinent information than a handover between two care providers in a primary care clinic. Because our instrument is not contingent upon a specific type of handover, it applies to various types of handovers by simply exchanging the SBAR tool for one that is applicable to the given context. Fourth, our instrument has the potential to be employed during real-time ratings as well as during video review. Being flexible to different modalities is advantageous because the availability of resources fluctuates. In other words, it may not be possible to have raters available to attend and observe every educational session; conversely, it may not be possible to have raters review numerous hours of video footage. Similarly, raters can select their modality based on convenience; therefore, it is beneficial to have an instrument that can be adapted for different modalities. Fifth, our instrument has the potential to be utilized by raters with varying skill levels. To expand, raters with extensive medical expertise (e.g., attending physicians) may be cost-intensive and often have limited time to dedicate to such tasks. Meanwhile, raters with less medical expertise (e.g., residents or interns) are more cost-effective and have different time demands. Finally, because this instrument only has three categories for ratings, it has the potential to require less training compared to more sophisticated ratings. Even though less sophistication would seemingly appear to be disadvantageous, such instruments require extensive training to achieve appropriate inter-rater reliability. Less sophisticated instruments, although not able to be as diagnostic as their more sophisticated counterparts, also necessitate less resources for training. Considering that many raters often have clinical or administrative responsibilities, having an instrument that potentially needs less training can be especially worthwhile. Further simplification, if desired, could bring the assessment to a dichotomous yes/no rating that could be utilized as a pass/fail assessment.

We posit that our instrument has merit; however, our study does have some limitations that are worth noting. First, while we analyzed a substantial number of handovers, this was an early phase, single institutional experience which limits its generalizability. Despite it being only a single experience, we would argue that such an event is necessary for establishing some of the psychometric properties of measurement instruments before a large-scale roll out becomes integrated into the curriculum. In addition, while we attempted to include a larger number of interprofessional students, only 25 health professional students versus 229 medical students participated. Therefore, this may impact the appropriateness of the use of this assessment instrument outside of the medical student population. Furthermore, when considering which form of structured communication framework to utilize, we selected SBAR due to its prevalence as well as its simplicity relative to other communication frameworks such as Illness Severity- Patient Summary-Action List-Situation Awareness and Contingency Planning- Synthesis by Receiver (IPASS) [61]. Given the inexperience of preclinical students and our goal to avoid cognitive overload of learners and raters, we felt the SBAR format met multiple needs. Our instrument is currently limited to the application of SBAR only; however, the overarching competency is ‘structured communication’, so the instrument could be modified such that structured communication represents a different handover framework (e.g., IPASS). Finally, there is room for improvement in this instrument regarding its diagnosticity and specificity. For example, there could be a greater number of assessment items for each competency. As another example, there could be greater granularity about the quality of the behaviors exhibited. Adding more items or categories would likely strengthen the diagnosticity of the instrument, but it can diminish the simplicity and usability of the instrument. A simplistic and usable instrument is advantageous for raters compared to more complicated instruments as it reduces the cognitive load required by raters. Complicated instruments can be cumbersome to use, are more cognitively taxing, and require more extensive training.

Given some of these limitations, we present several avenues for future research. Our study primarily focused on reliability, so one avenue future work should investigate additional types of validity. Another direction is to expand beyond a single educational experience and investigate handovers and competence longitudinally. A third idea is to examine additional student samples outside of medical students. All health professionals conduct handovers, so all of them need to perform handovers effectively. Consequently, all health professional students should be assessed accordingly. A fourth area for future expansion would be to modify the structured communication component to other handover frameworks (e.g., IPASS). Even though there have been mandates to strengthen handover standardization, handovers remain heterogeneous. Therefore, there would be utility in investigating structured communication within other handover frameworks. Finally, future work could examine additional items or even categories to strengthen the diagnosticity and granularity of the instrument and its reliability in the hands of evaluators with even more varied clinical expertise. Because handovers rely on safe behaviors, diagnostic insights into how to perform and improve handovers would be informative. That is, it is insufficient to simply do handovers, they must be performed safely and effectively.

## Conclusions

Handovers remain to be frequent, yet problematic events that necessitate multiple competencies to perform effectively. To determine if individuals are competent in conducting handovers, they must be assessed appropriately. Assessment instruments, therefore, need to demonstrate reliability and validity. The assessment instrument described in this study demonstrated substantial agreement, acceptable reliability, and preliminary construct validity in assessing competencies required for safe patient handover in pre-clinical learners. Further development of this instrument could be helpful in assessing entrustable professional activities (EPA #8) and Interprofessional Education Collaborative (IPEC) sub-competencies (Teamwork & Communication) in undergraduate medical and health professions students.

## Data Availability

The datasets (gradings from rater training and full assessment) used and analyzed during this study are available from the corresponding author on request. The student-led handover video recordings are not publicly available due to FERPA and are only available on reasonable request with permission of UT Southwestern.

## Abbreviations

AAMC: Association of American Medical Colleges
EPAs: Entrustable Professional Activities
SBAR: Situation-Background-Assessment-Recommendation
IPASS: Illness Severity- Patient Summary-Action List-Situation awareness and Contingency Planning- Synthesis by receiver
IPEC: Interprofessional Education Collaborative
